# Urinary Exosomes: A Novel Means to Non-Invasively Assess Changes in Renal Gene and Protein Expression

**DOI:** 10.1371/journal.pone.0109631

**Published:** 2014-10-13

**Authors:** Silvia Spanu, Claudia R. C. van Roeyen, Bernd Denecke, Jürgen Floege, Anja S. Mühlfeld

**Affiliations:** 1 Department of Nephrology, University of Medicine and Pharmacy “I. Hatieganu”, Cluj-Napoca, Romania; 2 Division of Nephrology and Immunology, Uniklinik RWTH Aachen, Aachen, Germany; 3 IZKF, Uniklinik RWTH Aachen, Aachen, Germany; INSERM, France

## Abstract

**Background:**

In clinical practice, there is a lack of markers for the non-invasive diagnosis and follow-up of kidney disease. Exosomes are membrane vesicles, which are secreted from their cells of origin into surrounding body fluids and contain proteins and mRNA which are protected from digestive enzymes by a cell membrane.

**Methods:**

Toxic podocyte damage was induced by puromycin aminonucleoside in rats (PAN). Urinary exosomes were isolated by ultracentrifugation at different time points during the disease. Exosomal mRNA was isolated, amplified, and the mRNA species were globally assessed by gene array analysis. Tissue-specific gene and protein expression was assessed by RT-qPCR analysis and immunohistochemistry.

**Results:**

Gene array analysis of mRNA isolated from urinary exosomes revealed cystatin C mRNA as one of the most highly regulated genes. Its gene expression increased 7.5-fold by day 5 and remained high with a 1.9-fold increase until day 10. This was paralleled by a 2-fold increase in cystatin C mRNA expression in the renal cortex. Protein expression in the kidneys also dramatically increased with de novo expression of cystatin C in glomerular podocytes in parts of the proximal tubule and the renal medulla. Urinary excretion of cystatin C increased approximately 2-fold.

**Conclusion:**

In this proof-of-concept study, we could demonstrate that changes in urinary exosomal cystatin C mRNA expression are representative of changes in renal mRNA and protein expression. Because cells lining the urinary tract produce urinary exosomal cystatin C mRNA, it might be a more specific marker of renal damage than glomerular-filtered free cystatin C.

## Introduction

An early and specific diagnosis and evaluation of disease activity are crucial elements for the choice of treatment modality in renal disease, i.e. immunosuppression versus conservative treatment. To date, the gold standard for diagnosis of renal disease is still a renal biopsy, an invasive diagnostic tool that is usually not suitable for follow-up diagnostics. Other diagnostic tools such as serum creatinine, microhematuria or proteinuria are either not sensitive enough (creatinine) or not specific for renal disease (microhematuria in urological disease or proteinuria in hypertension and cardiac insufficiency).

In the past, there have been numerous studies using urinary proteins as diagnostic markers for renal disease. Although there is a multitude of basic science papers, none of these markers has been translated into clinical practice [1]. This might be due to the underlying problem that proteins in the urine usually exist in low quantities (e.g. nephrin, podocin), are often reabsorbed in the tubular system or are subjected to proteolytic digestion. Similar problems exist for the evaluation of urinary mRNA as makers for renal disease [2]. A group from our own department was able to demonstrate that live podocytes detach during glomerular disease and can be cultured from the urine [3]. Data from animal models of glomerular disease showed that podocyturia is limited to phases of ongoing glomerular damage and might therefore be a more sensitive marker to assess the activity of glomerular disease. However, we and others have not been able to simplify and standardize the method to allow for translation into clinical practice. Another downside of podocyturia as a marker for glomerular disease might also be the fact that only viable cells are being assessed, and it is therefore probable that the larger proportion of apoptotic cells is being neglected. Also, damage to other glomerular cells such as mesangial cells cannot be assessed.

Consequently, new strategies need to be developed to diagnose renal disease and follow up on disease activity in order to target treatment more specifically. For this purpose, exosomes might represent a new diagnostic tool. Exosomes are small (40–100 nm) secreted membrane vesicles that are formed by inward budding of endosomal membranes which are released from the cell by fusion of the multivesicular body with the cell membrane. They contain plasma, proteins and RNA of the cells of origin. Exosomes can be isolated from different body fluids such as saliva, plasma and urine by differential centrifugation or membrane separation [4]; [5]. Their function, as known to date, may be in cell-to-cell communication and intercellular protein and RNA exchange [6]–[8]. Their main advantage seems to be the relative stability of these microparticles to proteinases and RNases [9]. Since our own work and data from others [10] have shown previously that protein lysates from urinary exosomes are contaminated by other urinary proteins, namely Tamm-Horsfall protein, we concentrated our efforts on establishing urinary exosomes as biomarkers for renal disease on the RNA level.

## Methods and Materials

### Puromycin aminonucleoside nephrosis

All animal experiments were approved by the Landesamt für Natur, Umwelt und Verbraucherschutz Nordrhein Westfalen. Animals were held in rooms with constant temperature and humidity and 12 h/12 h light cycles. Puromycin aminonucleoside nephrosis (PAN) was induced by i.p. injection of 150 mg/kg body weight puromycin (Sigma Aldrich, Saint Louis, USA) into male Sprague Dawley rats (n = 8 per group). 16-h urine samples were collected in metabolic cages at days 0 (before treatment), 5 and 10. Kidney function was assessed by measuring urinary protein, serum creatinine and urea levels and urinary cystatin C using an autoanalyzer. At the end of the experiment, animals were euthanized by lethal anesthesia with ketamine/rompun in addition to exsanguinations.

### Exosome isolation

Exosomes were isolated by differential centrifugation as described by Pisitkun et al. [11]. Briefly, urine samples were centrifuged at 17.000 g for 15 min at 4°C to remove urinary sediment. The supernatant was then centrifuged at 200.000 g for 45 min at 4°C (Optima L-80 XP, Beckman Coulter, Krefeld, Germany) to obtain exosomes. The pellets were resuspended in isolation buffer (10 mM triethanolamine/250 mM sucrose, pH 7.6 containing 0.5 mMPMSF and 1 µM leupeptin) or RLT buffer plus ß-mercaptoethanol for RNA isolation.

### Electron microscopy

The exosome pellet, resuspended in isolation solution was mixed 1∶2 with 3% glutaraldehyde. The suspension was applied to a 200 mesh nickel grid and scanned using a Philips EM 400T/ST electron microscope.

### RNA extraction, amplification and gene array analysis

For exosome mRNA analysis, total RNA (n = 3) was isolated from exosomal pellets using the RNeasy Mini Kit including the DNase digestion step (Qiagen, Hilden, Germany). Subsequently, two rounds of amplifications were carried out with the Riboamp HS Amplification Kit according to the manufacturer's instructions. RNA quantity was assessed using the NanoDrop 1000 (Thermo Scientific Nano Drop Technologies, Wilmington, DE, USA). Probes for the GeneChip RatGene 1.0 ST Array (each 300 ng) were prepared according to the Ambion WT Expression Kit (Ambion, Kaufungen, Germany) and the Affymetrix GeneChip WT Terminal Labeling Kit (Affymetrix, Santa Clara, CA, USA) manuals. The fragmented labeled sample was hybridized to an Affymetrix GeneChip RatGene 1.0 ST Array at 45°C for 16 hours at 60 rpm. Hybridised arrays were then washed and stained on Fluidics Station 450 (protocol FS_450_00007) and scanned on a GeneChip Scanner 3000 7G (both Affymetrix). The image data were analyzed with GeneChip Command Console Software (Affymetrix). Cell intensity files were processed by robust multiarray averaging [12] using Affymetrix Power Tools, distributed through AltAnalyze [13], using constitutive probe sets with detection above background p-values <0.01 and a raw expression threshold of 50. Threefold changes with a p-value of ≤0.01 were used as cut-off for up/down regulation.

### Quantitative RT-qPCR

Total RNA was extracted from exosomal fractions (n = 5) and whole cortex (n = 8) using the RNeasy kit from Qiagen (Qiagen, Hilden, Germany). For the analysis of exosomal RNA, mRNA-specific amplification was performed using the Arcturus RiboAmp HS PLUS Kit (Life Technologies, Paisley, UK) prior to real-time quantitative reverse transcriptase PCRs (RT-qPCR). cDNA synthesis and RT-qPCRs were performed as described earlier using the qPCR Core Kit for Sybr Green I (Eurogentec, Seraing, Belgium) and a 7300 real-time PCR machine (Applied Biosystems, Weiterstadt, Germany) [14]. Sequences of primers used for PCR are listed in Table 1. Each sample was normalized to the expression of the reference gene cyclophilin A. Cyclophilin A was chosen because of its high expression of urinary exosomes without significant changes during the course of PAN as demonstrated in the gene array analysis.

**Table 1:** Sequences of primers used for real-time reverse transcriptase PCR.

Gene	Forward primer	Reverse primer
Cystatin C	5′-TTCCAGCCACAAGCTGCTTA-3′	5′-CAACAAGGGCAGCAACGAT-3′
Cyclophilin A	5′-TGCTCATGCCTTCTTTCACCTT-3′	5′-TTATCTGCACTGCCAAGACTGAGT-3′

### Immunohistochemistry

Four-micrometer sections of methyl-Carnoy fixed, paraffin-embedded tissue sections were immunostained as described previously [15]. Cystatin C antibody was obtained from Abcam (ab109508; Abcam, Cambridge, UK). Negative controls for the immunohistochemical procedures consisted of substitution of the primary antibody with nonspecific IgG.

### Statistical analysis

Statistical analyses used for gene expression analysis arrays are described in the corresponding section. All remaining data were analyzed using the software Graph Pad Prism 6 for Windows (SPSS Inc., Chicago, IL). Results are presented as mean ± SD. Groups were compared with Kruskal-Wallis test followed by Dunn’s multiple comparison test. A probability (p) value <0.05 was considered statistically significant.

## Results

### Kidney function during the course of PAN

**Figure 1:**
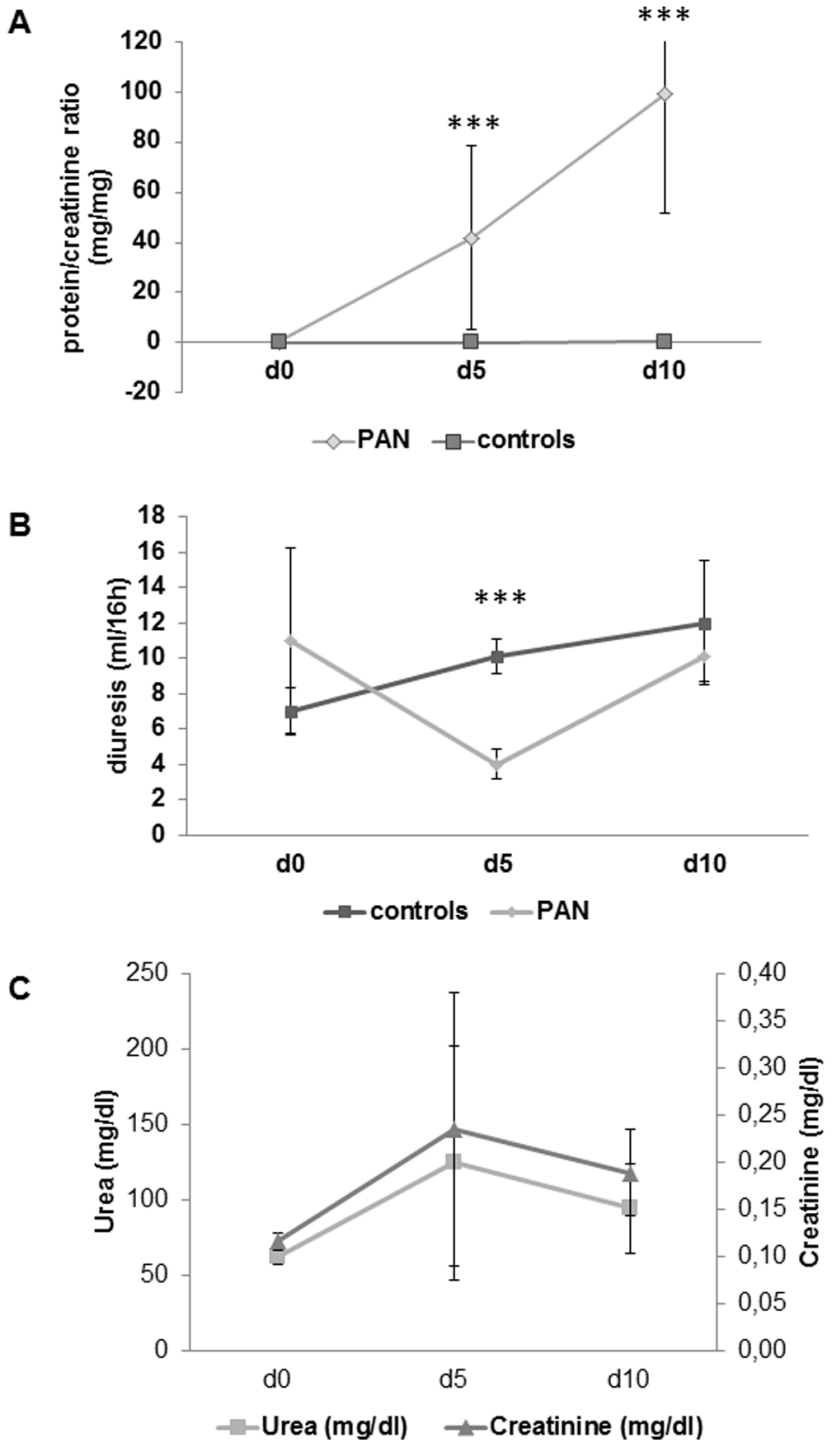
Kidney function during the course of PAN. A: Development of massive proteinuria 5 days after induction of PAN as indicated by an increase in protein/creatinine ratio. B: Decrease in urine output 5 days after the induction of PAN. C: Urea and creatinine on days 0, 5 and 10 after induction of PAN. Values represent mean ± SD. Statistical significant differences: *** p<0.001.

Animals developed massive proteinuria after induction of PAN starting at day 5 with an increase of protein/creatinine ratio from 0.1 mg/mg to 41.9 mg/mg (p<0.001). Urinary protein excretion increased further up until day 10 to 99.2 mg/mg (p<0.001). In addition to the development of proteinuria, rats with PAN developed pronounced oliguria (decrease in urinary output from 11 ml/16 hrs to 4 ml/16 hrs; p<0.001) five days after disease induction. Urinary output returned to baseline levels on day 10 (8 ml/16 hrs). Although there was a numerical increase in both serum creatinine and urea after 5 days into PAN, the changes were not statistically significant (Figure 1).

### Exosomal gene expression

Urine from rats at day 0 (before disease induction), day 5 after induction of puromycin aminonucleoside nephrosis and on day 10 of the disease was collected in metabolic cages over a time frame of 16 hours. Exosomes were isolated from the collected urine and presence and typical morphology of the exosomes was confirmed by electron microscopy. The mRNA isolated from the exosomal fraction of the urine was then subjected to gene array analysis. The most highly regulated genes in urinary exosomes during the course of PAN are shown in Table 2.

**Table 2:** Results of gene array analysis in urinary exosomes of rats during the course of PAN.

a		gene expression (log)			change in gene expression (log)	
gene	gene name	d0	d5	d10	d5/d0	d10/d5
**ND4L**	mitochondrially encoded NADH dehydrogenase 4L	5.0	9.8	7.2	4.8	–2.6
**Ptprs**	protein tyrosine phosphatase, receptor type, S	6.9	10.5	8.0	3.6	–2.5
**ND5**	mitochondrially encoded NADH dehydrogenase 5	5.3	8.5	6.8	3.2	–1.7
**ND6**	mitochondrially encoded NADH dehydrogenase 6	7.3	10.5	8.2	3.2	–2.3
**ATP8**	ATPase, aminophospholipid transporter, class I, type 8B	7.5	10.6	9.4	3.0	–1.1
**Cst3**	cystatin C	6.5	9.4	7.6	2.8	–1.7
**Tuba1a**	tubulin, alpha 1a	6.0	9.5	8.7	2.6	–0.8
**Spink1**	serine peptidase inhibitor, Kazal type 1	4.4	7.0	7.7	2.6	0.7
**COX1**	cyclooxygenase 1	7.3	9.4	7.7	2.1	–1.7
**Vim**	vimentin	5.7	7.7	6.7	2.0	–1.0
**Timp1**	TIMP metallopeptidase inhibitor 1	4.9	6.9	5.5	2.0	–1.4
**b**		**gene expression (log)**			**change in gene expression (log)**	
**gene**	**gene name**	**d0**	**d5**	**d10**	**d5/d0**	**d10/d5**
**Krt8**	keratin 8	5.9	4.9	4.3	–1.0	–0.6
**Fam3d**	family with sequence similarity 3, member D	6.0	4.9	4.5	–1.1	–0.5
**Map1lc3b**	Microtubule-associated protein 1 light chain 3 beta	7.9	6.8	5.0	–1.1	–1.8
**Pdcd4**	programmed cell death 4	6.9	5.8	5.3	–1.2	–0.5
**Vkorc1**	vitamin K epoxide reductase complex, subunit 1	8.0	6.8	6.3	–1.2	–0.5
**Fabp1**	fatty acid binding protein 1, liver	6.5	5.2	4.5	–1.3	–0.7
**S100a5**	S100 calcium binding protein A5	7.2	5.8	5.2	–1.5	–0.6
**Sh3bgrl3**	SH3 domain binding glutamic acid-rich protein like 3	10.6	9.1	8.5	–1.5	–0.6
**Snurf**	SNRPN upstream reading frame	6.7	4.7	4.4	–2.0	–0.3
**Fxyd4**	FXYD domain containing ion transport regulator 4	10.6	8.6	6.4	–2.0	–2.2

Top 10 differentially regulated genes in urinary exosomes of rats during the course of PAN. Gene expression is presented in log intervals of relative gene expression on the different days of PAN (d0, d5 and d10). In addition, the table shows changes in gene expression from day 0 to day 5 and from day 5 to day 10 also expressed in log intervals. Table 2 (a) shows the genes with the highest increase in expression during the course of PAN while (b) depicts the top 10 genes with reduced expression in urinary exosomes.

**Figure 2:**
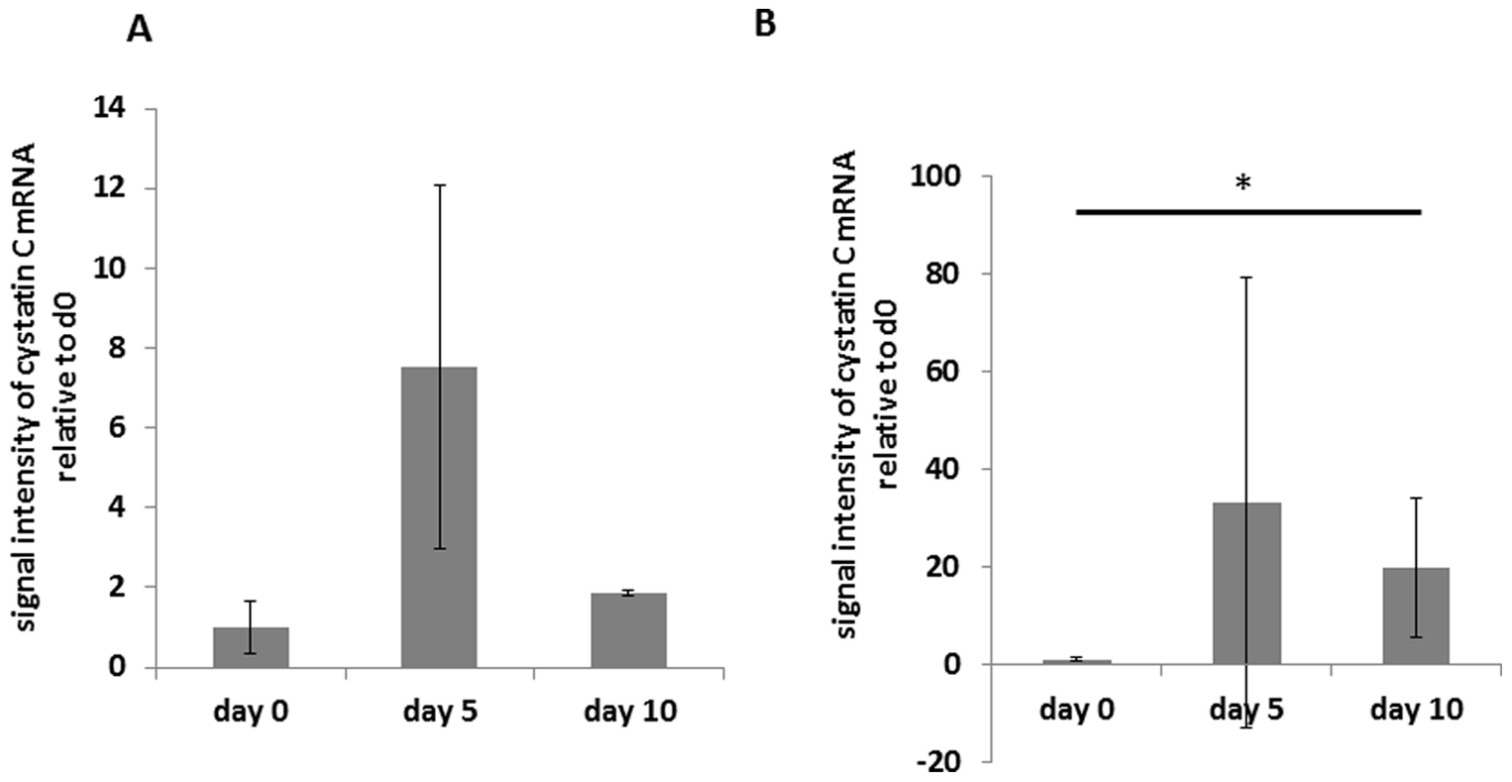
Exosomal gene expression of cystatin C mRNA. mRNA expression analysis in urinary exosomes isolated from the urine of rats during the course of PAN (day 0 before disease induction, day 5 after injection of puromycin and day 10 after disease induction). Cyclophilin A was used as reference gene. A: Cystatin C mRNA expression in gene array analysis from urinary exosomes relative to exosomes isolated from day 0, n = 3 animals. Bars represent mean ± SD. Significance level was assessed by Affymetrix Power Tools, using constitutive probe sets with detection above background p-values <0.01 and a raw expression threshold of 50. Threefold changes with a p-value of ≤0.01 were used as cut-off for up/down regulation. B: cystatin C mRNA from urinary exosomes relative to exosomes isolated from day 0 measured by RT-qPCR in separate animals, n = 3 animals. Bars represent mean ± SD. Statistical significant differences: *p<0.05.

Cystatin C was identified as a gene with a high baseline expression and significant changes during the course of the disease. Urinary exosomal cystatin C increased seven-fold from baseline (day 0) until day 5 of the disease. On day 10, it had decreased to a level almost twice the value of healthy animals (p<0.01). Quantitative RT-PCR for cystatin C using RNA isolated from urinary exosomes of different animals confirmed these results (Figure 2; p<0.05).

### Correlation of exosomal gene expression with mRNA and protein expression in the renal cortex

**Figure 3:**
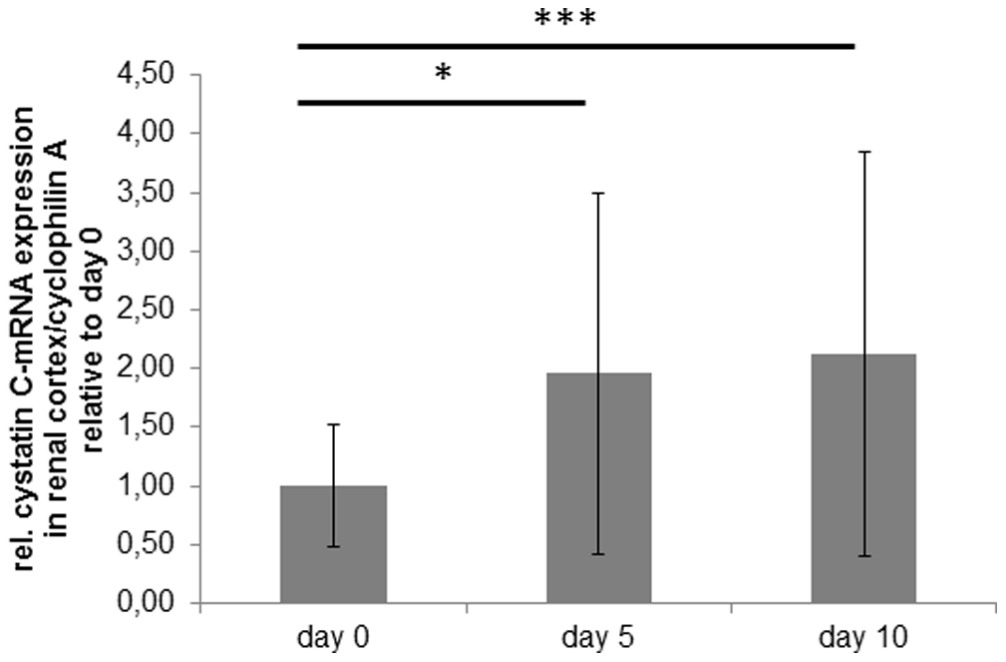
Cystatin C mRNA expression in the renal cortex. Cystatin C mRNA expression in lysates of the renal cortex of rats with PAN during the course of the disease (day 0, day 5 and day 10 after injection of puromycin). Results are expressed as rel. expression of cystatin c mRNA normalized to cyclophillin A relative to the means of day 0 animals. Bars represent mean ± SD. Statistical significant differences: * p<0.05; *** p<0.001.

Quantitative RT-PCR for cystatin C was performed on RNA isolated from the renal cortex of animals with and without PAN at different time points of the disease. Cyclophilin A was used as a reference gene as gene array analysis showed constant expression during the course of PAN. Cystatin C mRNA expression in the renal cortex doubled on day 5 after the induction of PAN and remained high at a 2.1 fold increase on day 10 compared to baseline (Figure 3).

**Figure 4:**
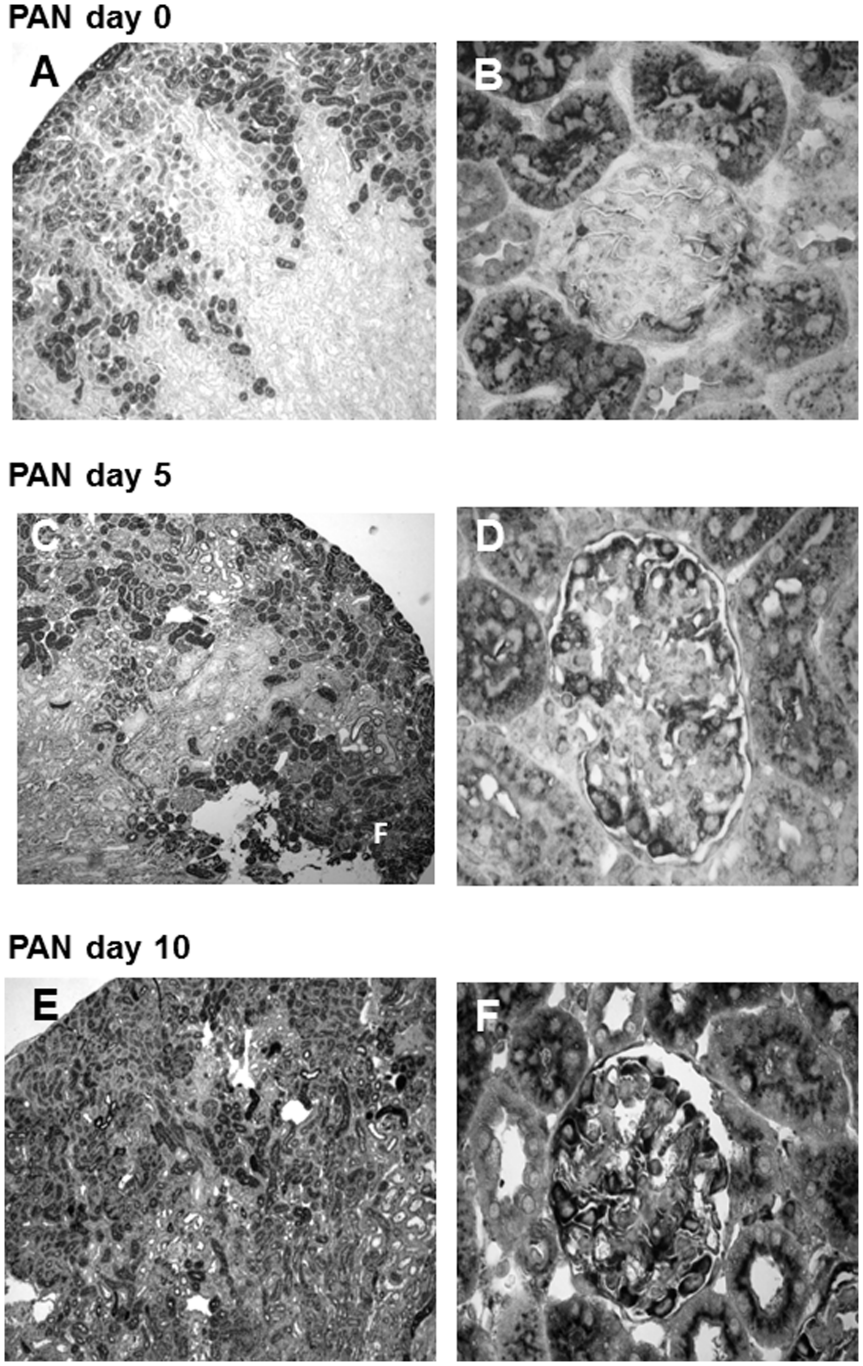
Immunohistochemistry staining of cystatin C in renal cortex of rats with PAN. A, C and E: Magnification 40-fold; B, D and F: magnification 400-fold; sections counterstained with methylen green. A+B: Cortex day 0, staining of the prox. tubules and minimal staining of the glomerular podocytes. C+D: PAN day 5, staining of additional segments of the proximal tubule as well as increased podocyte staining. E+F: PAN day 10, tubular staining similar to day 5, persistent cystatin C staining in podocytes.

In healthy rats without kidney disease, cystatin C was expressed in a granular cytoplasmatic fashion in cells of the proximal tubule. In glomeruli, it showed modest cytoplasmatic expression in some podocytes (Figure 4, A and B). After induction of kidney damage by puromycin, there was a strong increase in cystatin C staining in the proximal tubules as well as *de novo* staining in additional tubular segments (Figure 4, C). Tubular epithelium in the medulla also stained positive for cystatin C. Cystatin C staining could also be seen in proteinaceous material within the tubular lumen. In addition, there was a pronounced increase in podocyte-specific staining in the glomerulus (Figure 4, D). On day 10, immunohistochemical staining for cystatin C remained similar to the staining pattern on day 5 after induction of PAN, and only the podocyte-specific staining seemed to be more pronounced (Figure 4, E and F).

### Urinary cystatin C excretion

**Figure 5:**
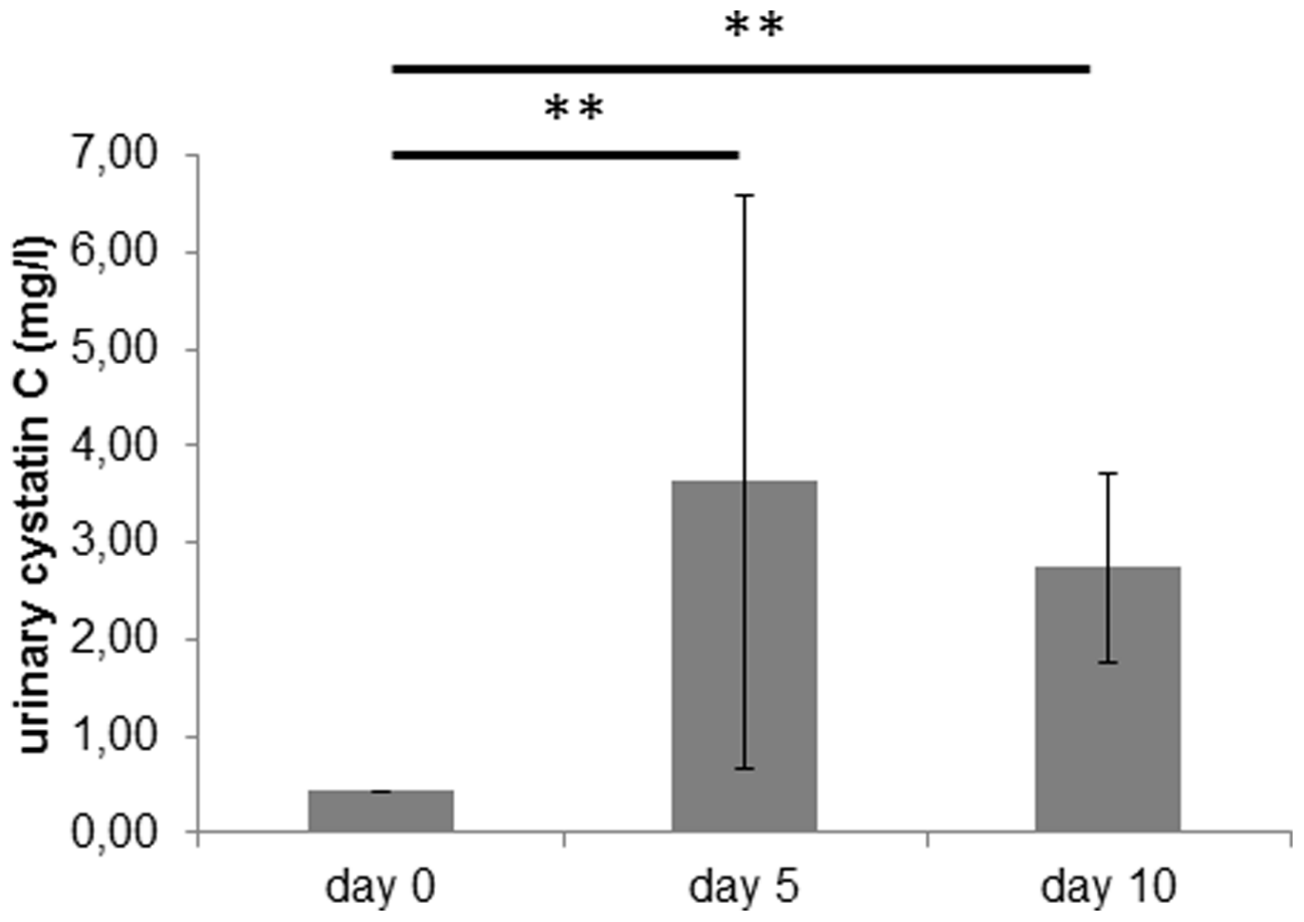
Urinary cystatin C expression. Cystatin C excretion in the urine (mg/l) of rats with PAN on the different days after induction of PAN. Bars represent mean ± SD. Statistical significant differences: ** p<0.01.

Urinary excretion of cystatin C was low at 0.4±0.0 mg/l in healthy rats and increased to 3.6±3.0 mg/l at day 5 of PAN (p<0.01). On day 10, it was significantly increased compared to baseline at 2.7±1.0 mg/l (p<0.01) (see Figure 5).

## Discussion

In this pilot study, we assessed the use of urinary exosomes as a biomarker during the course of puromycin aminonucleoside nephrosis (PAN), in an animal model mimicking podocyte damage in minimal-change glomerulonephritis. Exosomes are manufactured in an active process within the multivesicular endosomes (MVE) and secreted into various kinds of body fluids by fusion of the MVE with the plasma membrane [16]. They contain proteins and RNA of their cell of origin. In contrast to free urinary proteins or RNA, exosomal contents are protected against exogenous proteinases and RNases by their surrounding plasma membrane [9]. Exosomes in the urine originate mainly from cells lining the urinary tract including bladder, prostate gland, renal collecting ducts and proximal tubules. However, exosomes from distant anatomic sites, such as the liver, have also been found in the urine [17]. The availability of large quantities of urine and the non-invasive nature of the test seems to make them an ideal tool for the diagnosis and follow-up of kidney disease.

Gonzales et al. have performed proteomic analysis of urinary exosomes and identified over 1100 different proteins [18]. Further analysis of the exosomal proteome revealed fetuin A as a potential biomarker for acute kidney injury (AKI) [19]. Other differentially expressed proteins identified by mass spectrometry in the exosomal proteome could not be verified by western blot analysis. This might be due to the relatively low amount of exosomal protein in the urine that might allow protein detection with the very sensitive tool of mass spectrometry while the detection threshold of western blotting might not be sensitive enough for the identification of individual proteins. A second point that might negatively influence the utility of exosomal urinary proteins as a biomarker for kidney disease might be the fact that urinary proteins, especially Tamm-Horsfall protein, stick to the outer surface of exosomes, reducing the exosome yield and potentially contaminating exosomal proteins from the outside [10].

Despite these drawbacks, some groups assessed different proteins in urinary exosomes as biomarkers of kidney disease. Sonoda et al. found a significant reduction in urinary exosomal aquaporin-1 (AQP1) expression, a water channel protein expressed in renal epithelial cells of the proximal tubules and descending thin limb, in rats after ischemia-reperfusion injury (I/R). These changes in exosomal protein expression were accompanied by histological AQP1 protein retention in the early phase and decreased expression of AQP1 in tissue of the renal cortex in the later phase of I/R [20]. Esteva-Font et al. could demonstrate that urinary exosomal NKCC2 and NCC excretion rates correlated with their immunohistochemical abundance in the kidney [21]. However, in the clinical setting, both urinary exosomal proteins showed no correlation to tubular sodium reabsorption in hypertensive patients. In addition, WT1 protein expression in urinary exosomes has failed as a biomarker for childhood nephrotic syndrome [22]. The same marker was also explored in patients with diabetic kidney disease where WT1 expression in urinary exosomes was significantly higher in patients with proteinuria than those without [23] and in patients with FSGS where an association with the activity of the disease was shown [24].

So far, urinary exosomal RNA as biomarkers for kidney disease has only been assessed by a few groups. Lv et al. were able to demonstrate that exosomal CD2AP mRNA was lower in patients with kidney disease compared to healthy controls and that its expression decreased with increasing severity of proteinuria [25]. CD2AP mRNA correlated negatively with 24 h-urine protein, severity of tubulointerstitial fibrosis and glomerulosclerosis and could discriminate between kidney disease and controls. The same group identified urinary exosomal miRNA-29c as a biomarker which correlates with kidney function and renal fibrosis in patients with CKD [24]; [26]. A different group explored urinary exosomal miRNA in diabetic nephropathy. They were able to show that miR145 expression was altered in type 1 diabetic patients with incipient diabetic nephropathy [27]. Gildea et al. could identifiy 45 urinary exosome miRNAs that were associated with an individual’s blood pressure response to sodium [28].

In our study, we found that exosomal mRNA content for cystatin C correlates with the disease activity of PAN as it is found in much higher expression in rats after the onset of proteinuria induced by podocyte damage. We were able to demonstrate that these changes in exosomal mRNA reflect changes in tissue-specific mRNA and protein expression, emphasizing their potential role as a non-invasive marker of intrarenal changes. This was shown despite the fact that the number of animals assessed in our study was small, resulting in a rather high standard deviation. Further studies with higher numbers of animals, maybe extending into different models of kidney disease are necessary to further assess the utitiliy of urinary exosomal mRNA as a marker of kidney disease.

In a recent study, Peake et al. assessed the urinary exosomal mRNA expression of neutrophil gelatinase-associated lipocalin (NGAL), interleukin-18 (IL 18), kidney injury molecule-1 (KIM-1) and cystatin C in the urine of patients after kidney transplantation and found no correlation of the exosomal mRNA levels with the 7 day creatinine reduction ratio (CRR 7) while the urinary proteins NGAL and IL-18 reflected the CRR at day 7 [29]. In contrast to our results, they found no correlation between exosomal cystatin C mRNA which remained stable after kidney transplantation and urinary cystatin C protein which showed a temporary increase.

In our model, Cystatin C mRNA was chosen as a marker because gene array analysis revealed that it is expressed in urinary exosomes of healthy rats in adequate amounts and showed marked upregulation after the induction of podocyte damage. Cystatin C is an active protease inhibitor and is found in high concentrations in all biological fluids. It is a low-molecular weight protein, which is produced constantly by all nucleated cells and is eliminated from the blood by glomerular filtration. After its filtration into the urine, it is reabsorbed and catabolized in the tubules with the remaining protein being eliminated in the urine [30]. In the kidney, it has been shown to be expressed in the proximal tubules and its expression pattern was not changed either in an experimental model of diabetic nephropathy [31] or in cisplatin-treated rats [32]. In our study, we could demonstrate staining in the proximal tubules in healthy rats while induction of PAN led to an extensive expansion of the tubular area staining positive for cystatin C as well as *de novo* expression of cystatin C in medullary tubular epithelial cells. In addition, the induction of podocyte damage by puromycin led to a *de novo* expression of cystatin C in the podocytes.

In clinical studies, free urinary cystatin C has been identified as a diagnostic marker for acute kidney injury (AKI) induced by sepsis [33], cardiac surgery [34] or drug toxicity [35]. Increased urinary cystatin C concentrations allow the accurate detection of tubular dysfunction of pure and mixed nephropathies [36]. Clinical test have shown that its utility as a marker for tubular injury is higher when it is not adjusted to urinary creatinine, improving the negative predictive value [37]. In addition to its use as a diagnostic marker for AKI, a type of kidney injury thought to be driven mainly by tubular injury, urinary cystatin C was also shown to predict progression of diabetic nephropathy [38]. When cystatin C was assessed as a marker of nephrotoxicity in animal models of puromycin- and doxorubicin-induced glomerular injury, it showed a better diagnostic performance for glomerular injury than BUN and serum creatinine [35]. This might be due to the fact that in kidney injury resulting in high proteinuria, urinary excretion of cystatin C has been shown to be increased [39] probably by blocking tubular reabsorption and degradation of cystatin C in the tubules [40]. Using urinary exosomal cystatin C mRNA content might potentially increase the sensitivity of urinary cystatin C as it is not degraded by urinary proteinases as it was shown for free cystatin C (6–12% of urinary cystatin C is degraded after 3 days at room temperature) [36]. In addition, it might be more specific for renal damage as the majority of urinary exosomes is probably not filtered from the circulation but produced in the kidney itself.

In conclusion, this “proof-of-concept” study demonstrates that urinary exosomal mRNA, as demonstrated using the example of cystatin C mRNA, reflects intrarenal changes in mRNA and protein expression and might thus be a good marker for either the diagnosis of intrinsic kidney disease or the follow-up of established CKD.
